# Exploring Anti-Inflammatory Treatment as Upstream Therapy in the Management of Atrial Fibrillation

**DOI:** 10.3390/jcm14030882

**Published:** 2025-01-29

**Authors:** Edward Zheng, Izabela Warchoł, Maja Mejza, Maria Możdżan, Monika Strzemińska, Anna Bajer, Paulina Madura, Juliusz Żak, Michał Plewka

**Affiliations:** Department of Interventional Cardiology and Heart Rhythm Diseases, Teaching Hospital No.2 of Medical University of Lodz, Faculty of Medicine, Medical University of Lodz, Zermoskiego 114, 91-647 Lodz, Poland; maja.mejza@student.umed.lodz.pl (M.M.); maria.mozdzan@student.umed.lodz.pl (M.M.); monika.strzeminska@student.umed.lodz.pl (M.S.); anna.bajer1@student.umed.lodz.pl (A.B.); paulina.madura@student.umed.lodz.pl (P.M.); juliusz.zak@student.umed.lodz.pl (J.Ż.); michal.plewka@umed.lodz.pl (M.P.)

**Keywords:** anti-inflammatory treatment, atrial fibrillation, fibrosis, inflammation

## Abstract

Inflammation has been widely recognized as one of the major pathophysiological drivers of the development of atrial fibrillation (AF), which works in tandem with other risk factors of AF including obesity, diabetes, hypertension, and heart failure (HF). Our current understanding of the role of inflammation in the natural history of AF remains elusive; however, several key players, including the NLRP3 (NLR family pyrin domain containing 3) inflammasome, have been acknowledged to be heavily influential on chronic inflammation in the atrial myocardium, which leads to fibrosis and eventual degradation of its electrical function. Nevertheless, our current methods of pharmacological modalities with reported immunomodulatory properties, including well-established classes of drugs e.g., drugs targeting the renin–angiotensin–aldosterone system (RAAS), statins, and vitamin D, have proven effective in reducing the overall risk of developing AF, the onset of postoperative atrial fibrillation (POAF), and reducing overall mortality among patients with AF. This might bring hope for further progress in developing new treatment modalities targeting cellular checkpoints of the NLRP3 inflammasome pathway, or revisiting other well-known anti-inflammatory drugs e.g., colchicine, vitamin C, nonsteroidal anti-inflammatory drugs (NSAIDs), glucocorticosteroids, and antimalarial drugs. In our review, we aim to find relevant upstream anti-inflammatory treatment methods for the management of AF and present the most current real-world evidence of their clinical utility.

## 1. Introduction

Atrial fibrillation (AF) is a supraventricular tachyarrhythmia, characterized by an atrial uncoordinated electrical activation, which leads to a non-effective atrial contraction [1,2] and increased risk of thrombus formation within the chamber of the atrium. It is the most common arrhythmia, affecting around 40 million people worldwide [3]. Its incidence positively correlates with age. Consequently, due to generally extended life expectancy, it is predicted to increase over twice [4].

This condition may result in various symptoms, such as palpitations, impaired exercise tolerance, tiredness, fainting, and dizziness, contributing to a reduced quality of life. However, AF may be asymptomatic and, in those cases, is often diagnosed after the occurrence of complications, such as stroke or heart failure (HF) [5]. Patients with AF are five times more at risk of cardiovascular complications, and therefore, numerous methods of prevention are introduced [6]. Anticoagulant medication is recommended, as well as left atrial appendage occlusion. However, these methods have their limitations [7]. Therefore, it is essential to understand the association between AF and inflammation to introduce new therapeutic options.

The most common risk factors predisposing the development of AF are advanced age and male sex. Modifiable risk factors include low physical activity, smoking, obesity, hypertension, diabetes, and HF, which are conditions associated with an increased inflammatory state [8]. Moreover, atrial tissue’s electrical abnormalities and structural remodeling are linked to AF development [9]. In recent years, the influence of immune factors in the development of AF has also been studied [10].

**Figure 1:**
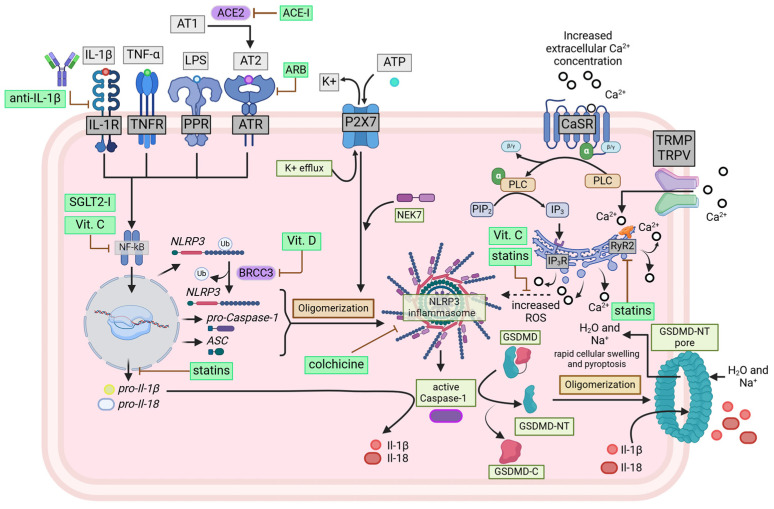
Overview of the activation of the NLRP3 inflammasome and its consequences on the myocyte, adapted from Kelley et al. [12] and Dobrev D et al. [11]. The NLRP3 inflammasome is a key component of chronic inflammation, which drives cardiac fibrosis and further loss of function. As pointed out, there are pharmacological modalities which can attenuate its activation through targeted inhibition of its checkpoints. NLRP3—NLR family pyrin domain containing 3, NF-κB—nuclear factor kappa-light-chain-enhancer of activated B cells, BRCC36—Lys-63-specific deubiquitinase 36, ASC—apoptosis-associated speck-like protein containing a caspase recruitment domain, TNF-α—tumor necrosis factor-α, TNFR—tumor necrosis factor-α receptor, PPR—pattern recognition receptors, LPS—lipopolysaccharide, AT2—angiotensin 2, AT2R—angiotensin 2 receptor, ACE2—angiotensin-converting enzyme 2, ARB—angiotensin II receptor blockers, ATP—adenosine triphosphate, NEK7—NIMA-related kinase 7, PIP2—phosphatidylinositol 4,5-bisphosphate, PLC—phospholipase C, ROS—reactive oxygen species, Il-1B—interleukin-1B, Il-18—interleukin-18, Il-1R—interleukin-1B receptor, SGLT2-I—Sodium–glucose Cotransporter-2 Inhibitors. Created with BioRender.com.

General and local inflammation processes are associated with the onset and maintenance of AF. Cardiomyocytes exhibit potentially pro-inflammatory signaling, which is controlled by the NLRP3 (NLR family pyrin domain containing 3) inflammasome [Figure 1] [11]. The inflammasome seems to be involved in arrhythmogenesis by enhancing RyR2-mediated Ca^2+^ release by the sarcoplasmic reticulum. Moreover, activation of the NLRP3 inflammasome promotes the formation of IL-1β and IL-18 [12]. Also, an increase in high-sensitivity C-reactive protein level is shown to occur with inflammasome activation in AF patients [13].

## 2. The Relationship Between Inflammation and AF

The role of inflammation in the pathogenesis and maintenance of atrial fibrillation is a key focus of various research. Delving into the potent role of inflammatory markers in AF, scientists noted the increased risk of AF in different inflammatory diseases [14]. This type of arrhythmia is commonly seen as a complication of pericarditis, myocarditis, and endocarditis [15]. While a cause-and-effect relationship between AF and inflammation has not been established, there is strong evidence of an association between these conditions. The belief is that stagnant blood flow in AF leads to endothelial microinjury in the endocardium, facilitating the migration of immune cells into the atrial tissue [16]. This leads to leukocyte infiltration and subsequent electrical and structural remodeling in the atrium. Moreover, there is substantial evidence of the relationship between AF and both local and systemic inflammation, as indicated by biomarkers and the anti-arrhythmic effects of anti-inflammatory therapy [17].

**Figure 2:**
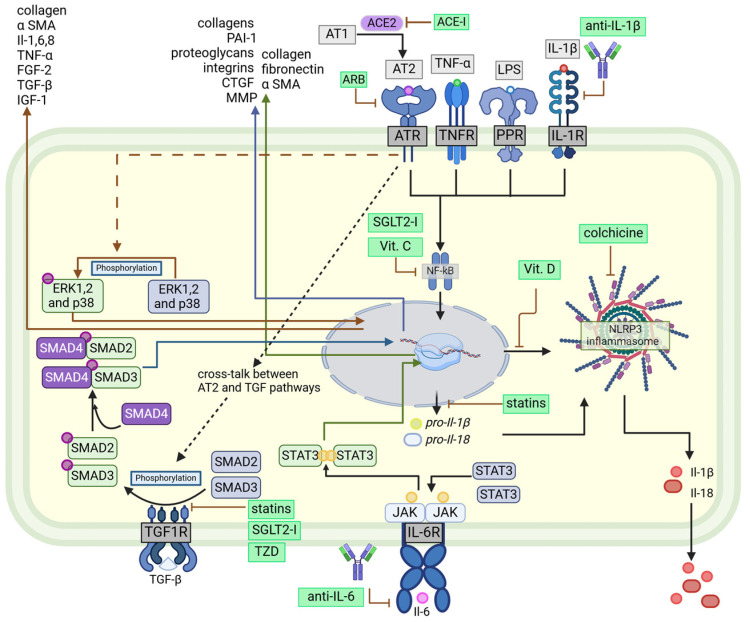
Overview of the role of inflammatory cytokines and pathways on cardiac fibroblasts which stimulates their proliferation, transformation into the myofibroblast, and excessive synthesis of extracellular matrix components such as collagen, proteoglycans, integrins and fibronectin, pro-inflammatory cytokines (Il-1B, 6, 8, 18, TNF-a), and growth factors including CTGF (connective tissue growth factor), FGF-2 (fibroblast growth factor-2), TGF-β (transforming growth factor β), and IGF-1 (insulin-like growth factor 1). Figure adapted from Turner N et al. [18]. Il-6R—interleukin-6 receptor, STAT3—Signal transducer and activator of transcription 3, JAK—Janus kinase, α-SMA—α Smooth Muscle Actin, NLRP3—NLR family pyrin domain containing 3, NF-κB—nuclear factor kappa-light-chain-enhancer of activated B cells, TNF-α—tumor necrosis factor α, TNFR—tumor necrosis factor-α receptor, PPR—pattern recognition receptors, LPS—lipopolysaccharide, AT2—angiotensin 2, ATR—angiotensin 2 receptor, ACE2—angiotensin-converting enzyme 2, ARB—angiotensin II receptor blockers, Il-1β—interleukin-1β, Il-18—interleukin-18, Il-1R—interleukin-1β receptor, TZD—thiazolidinediones, SGLT2-I—Sodium–glucose Cotransporter-2 Inhibitors, MMP—Matrix metalloproteinases, PAI-1—Plasminogen activator inhibitor-1, ERK—extracellular signal-regulated kinases. Created with BioRender.com.

Intercellular fibrosis plays a significant role in disrupting atrial conduction and function. This disruption of signal coordination across the atrial myocardium promotes the formation of re-entrant circuits and perpetuates the arrhythmic substrate, by the pathological remodeling of the atrial tissue, largely due to fibroblast activation and their differentiation into cardiac myofibroblasts, contributing to the maintenance and progression of AF [Figure 2]. Macrophages also have a similar impact, enhancing fibrosis by producing cytokines, such as IL-6 and TGF-β [10] [18]. The links between chronic inflammation and AF are further supported by the fact that, in AF patients, local atrial tissue demonstrated higher nuclear factor-kB (NF-kB) activity, a higher concentration of serum tumor necrosis factor-α (TNF-α) and IL-6, and a higher ECM volume than patients with sinus rhythm [19]. Other mediators associated with fibrosis and AF occurrence, such as platelet-derived growth factor and oxidative stress marker GDF-15, are linked to a higher risk of stroke and death [20].

Moreover, chronic inflammation can additionally lead to alterations in gene expression, e.g., TGF-β1 was shown to mediate the overexpression of genes associated with fibrosis via the phosphorylation of Smad2 [21]. Another genetic pathway leading to AF is via sodium, calcium, and potassium channel dysfunction [22]. Alterations in the first two are connected to increased fibrosis, whereas potassium level changes are mostly related to action potential shortening, which promotes the re-entry mechanism. It has been previously established that acute inflammation may influence these ion concentrations via interactions with ST2 [23], further highlighting the need for more studies on the subject.

Apart from structural and epigenetic changes, there are also electrical ones associated with pro-inflammatory states. Electrical remodeling in AF creates a self-perpetuating cycle that involves shortening the atrial refractory period and prolonging atrial conductivity [24]. Inflammation through angiotensin II influences NF-kB subunit overexpression, which dysregulates the Na channels in the atrium and contributes to the pro-arrhythmic effects [25]. Moreover, the angiotensin II receptor AT1 stimulates the interaction between transmembrane protein CD44, hyaluronic acid, and STAT3, thereby promoting fibrosis via increased expression of hyaluronic acid and collagen [26]. Another inflammatory cytokine, IL-6, reduces in vivo expression of connexins, which are proteins that bind myocytes end-to-end and form communication channels between cells [27] This disruption of connexin’s function affects the electrocardiographic P-wave indices [28].

The innate immune system also shows a relationship with AF. The inflammasome, a critical component of non-specific immunity, is a cytosolic multiprotein complex essential for the activation of interleukin-1β (IL-1β), a key mediator of the inflammatory response [29]. Inflammasomes come in different forms, such as NLRC4, NLRP1, and NLRP3, each associated with a specific NLR protein [16]. The NLRP3 inflammasome, called cryopyrin, can be activated through pattern recognition receptors (PRRs) by various external stimuli, including pathogens, glycolytic products, uric acid crystals, and ATP. It comprises three components: the NLRP3 sensor, the ASC adaptor, and the Caspase-1 effector [30]. The NLRP3 stimulation activates Caspase-1, which then processes the precursor forms of the pro-inflammatory cytokines IL-1β and IL-18 into their active forms [31]. Inflammasomes have been detected in atrial cardiomyocytes, and their signaling plays a role in the fibro-inflammatory process associated with AF. When activated, the NLRP3 inflammasome releases pro-inflammatory cytokines, creating a chronic inflammatory environment and promoting atrial remodeling and fibrosis. These changes disrupt the atrial tissue’s normal electrical conduction and structural integrity, increasing the susceptibility to AF [32]. Enhanced activation of NLRP3 has been observed in patients with AF and positively correlates with the progression of arrhythmias, atrial dilation, and structural remodeling. Mouse models with chronically active NLRP3 inflammasomes show greater susceptibility to AF, whereas mice with inhibited NLRP3 have a reduced risk of developing the condition [32]. Additionally, elevated levels of the pro-inflammatory cytokines IL-18 and IL-1β, which are also increased in AF patients, may result from excessive NLRP3 activation [33,34].

### 2.1. Obesity as a Pro-Inflammatory Risk Factor for Atrial Fibrillation

Both the latest 2024 ESC and 2023 ACC/AHA/ACCP/HRS guidelines highlight obesity (BMI > 30 kg/m^2^) and overweight (BMI > 25 kg/m^2^) as significant, modifiable pathogenic contributors to AF due to their correlation with a higher incidence of AF onset; this is mainly because of their role in maintaining chronic low-grade inflammation and their frequent association with other comorbidities that promote the development of AF substrate, such as hypertension, diabetes, and obstructive sleep apnea [35,36].

Evidence from population cohort studies has consistently demonstrated a positive correlation between increased BMI and AF incidence. In an observational analysis of the Framingham Heart Study, Wang TJ et al. [37] reported a 4% increase in AF risk for every 1% increase in BMI. Moreover, obesity has been identified as a notable risk factor for AF among younger, fertile women. A cohort study [38], conducted on the Danish female population, revealed a hazard ratio (HR) of 2.04 (95% CI: 1.13–3.69) for obese individuals and 3.50 (95% CI: 1.86–6.58) for severely obese individuals, compared to those of average weight, after adjusting for age, hyperthyroidism, and prior use of beta-blockers.

Concordant with these findings, current guidelines strongly recommend (Class 1B/1B-R) a weight reduction of greater than 10% in overweight or obese patients (BMI > 27 kg/m^2^) as part of a comprehensive risk factor management strategy to reduce AF-related symptoms, burden, recurrence, and progression. This recommendation is partially based on results from a randomized controlled trial (RCT) by Abed HS et al. [39], in which a 14.5% reduction in weight led to significant clinical improvements in AF patients, evidenced by a marked decrease in the Atrial Fibrillation Severity Scale (AFSS) compared to the control group (Δ 8.4 vs. 1.7, *p* = 0.001) and a substantial reduction in AF burden (*p* = 0.001). Similar outcomes were observed in the LEGACY study [40] (Long-Term Effect of Goal-directed Weight Management on Atrial Fibrillation Cohort: a 5-Year Follow-up Study), where patients achieving a weight reduction of more than 10% demonstrated significantly lower AFSS scores over five years compared to those with lesser weight loss. Additionally, patients in the >10% weight loss group had a sixfold higher probability of remaining arrhythmia-free (95% CI: 3.4 to 10.3, *p* < 0.001) than those in the other groups. Furthermore, weight loss has been shown to enhance the outcomes of catheter ablation. In the ARREST-AF trial [41], patients assigned to risk factor management, which included >10% weight reduction, had a lower rate of arrhythmia recurrence than the control group (67.1% vs. 90.3%, *p* < 0.001).

The pathophysiology underlying chronic low-grade inflammation in obese and overweight individuals is multifaceted, with visceral adiposity playing a pivotal role. Adipose tissue, especially visceral fat, exhibits both endocrine and immunological functions, secreting pro-inflammatory cytokines such as interleukins (IL-1β, IL-6, IL-8, IL-18) [42,43,44,45], leptin [46], tumor necrosis factor-α (TNF-α), monocyte chemoattractant protein-1 (MCP-1), and resistin [47]. Interleukins, particularly IL-1β, and TNF-α, are critical in activating the NLRP3 inflammasome, which drives cardiac remodeling. Additionally, adipocyte-induced inflammation contributes to an imbalance in the regulatory CD4+ T cells and effector CD8+ T cells, which promotes macrophage overactivation and amplifies the inflammatory cascade [48].

Excessive epicardial adipose tissue (EAT), which is anatomically adjacent to the atrial myocardium, may be instrumental in promoting local inflammation and cardiac remodeling. This is due to its role in the paracrine secretion of inflammatory mediators and its close anatomical and microcirculatory connection with the atrial myocardium [48]. EAT further fuels localized inflammatory responses and contributes to atrial remodeling [49].

### 2.2. Smoking as a Pro-Inflammatory Risk Factor for Atrial Fibrillation

In a meta-analysis of the risk of atrial fibrillation among smokers in Atherosclerosis Risk in Communities (ARIC) Studies [50], smokers, when compared to never-smokers, were significantly more prone to developing AF regardless of their current smoking status (former smokers (HR = 1.32 Cl = 95%, 1.10–1.57), current smokers (HR = 2.05 Cl = 95%, 1.71–2.47), ever smokers (HR = 1.58 Cl = 95%, 1.35–1.85)). Despite the proven initial correlation between smoking and a higher risk of AF onset, in a sub-analysis of the population cohort REGARDS (REasons for Geographic And Racial Differences in Stroke), after adjustment for cardiovascular risk factors, smoking did not reach statistical significance as a risk factor for the development of AF; this raises the question of whether AF stems from other cardiological conditions induced by smoking i.e., CAD (coronary artery disease) or if smoking can independently provoke AF onset. Nevertheless, population cohorts have congenitally shown that cessation of smoking can greatly benefit patients with AF, as smoking itself has also been associated with an increased risk of AF recurrence after PVI (pulmonary vein isolation) [51], a higher risk of dementia [52], stroke, CVD, and all-cause mortality among patients with AF [53]; this explains the rationale of the ACC/AHA/ACCP/HRS 2023 recommendation for smoking cessation among patients with a history of AF.

The primary mechanism by which smoking contributes to the inflammatory state is related to the exertion of oxidative stress by inhaled immunomodulatory particles such as nicotine, acrolein, and reactive oxidative substances (ROS), which activate further cellular cascades related to atherosclerosis, cardiac muscle dysfunction [54], and electrical aberrations within the myocardium [55].

The role of smoking in the promotion of systemic inflammation and its potential link to the pathogenesis of the AF substrate has been partially elucidated, as smoking increases serum levels of Il-18 and -1β over two- to sevenfold [56] when compared to non-smokers, which marks excessive NLRP3 inflammasome activation. Moreover, nicotine has been found to exert oxidative stress on the endothelium which additionally fuels NLRP3 inflammasome activation and pyroptosis [57], which might be a common link between CAD and AF pathogenesis among smokers.

### 2.3. Alcohol Consumption as a Pro-Inflammatory Risk Factor for Atrial Fibrillation

The relationship between alcohol consumption and AF is well-established. It has been emphasized in both the latest ACC/AHA/ACCP/HRS 2023 and ESC 2024 guidelines as a modifiable risk factor of AF. Alcohol is recognized as a significant risk factor for the onset, progression, and recurrence of AF after ablation [58]. Evidence from the retrospective analysis of the LEGACY trial [59] demonstrated that patients in the intervention group, who were advised to limit alcohol intake to below 30 g/week as part of a comprehensive weight loss and risk factor management strategy, exhibited reduced progression from paroxysmal to persistent AF. Furthermore, absolute abstinence from alcohol has consistently shown a protective effect against the onset of AF. In a nationwide cohort by Lee et al. [60], abstinence was associated with a 63% reduction in the incidence of AF onset compared to heavy drinkers. Similar findings were reported in another nationwide cohort by Kim et al. [61], which identified moderate and heavy drinking as independent risk factors for increased AF development (HR = 1.129; 95% CI = 1.097–1.161, HR = 1.298; 95% CI = 1.261–1.337, respectively). Supporting this, a meta-analysis by Jiang et al. [62] of 13 prospective studies concluded that even one drink per day was associated with a 6% increase in AF risk (RR: 1.06; 95% CI: 1.03–1.08).

Paradoxically, alcohol may exhibit anti-inflammatory effects on the myocardium through mechanisms such as attenuating the NLRP3 inflammasome. Animal studies have shown that alcohol can inhibit the activation of the NLRP3 inflammasome [63] and NFκB pathways [64], leading to a reduction in the secretion of pro-inflammatory cytokines. The consumption of red wine, in particular, has been associated with a cardioprotective effect against coronary artery disease (CAD) and has been suggested to possess anti-inflammatory properties. However, ethanol itself has not been conclusively shown to reduce systemic inflammation [65]. Despite its potential anti-inflammatory properties, alcohol is also widely reported to induce adverse cardiac remodeling. This includes increased myocardial fibrosis, electrophysiological alterations, and oxidative stress at the cellular level, which collectively contribute to the development of the AF substrate. This duality underscores the complex interplay of alcohol’s effects on cardiac health and the predominant evidence supporting its role as a risk factor for AF [66,67,68].

### 2.4. Chronic Infections and Their Relation to Atrial Fibrillation

Cardiovascular diseases, including AF, are more common among the population with HIV (PWH) compared to the general population [69]. This is particularly true for those with CD4 counts < 200 cells/mm^3^ [70]. The higher incidence of these conditions is likely linked to abnormal immune activation and chronic inflammation. Antiretroviral therapy (ART) is known to reduce inflammation [71], suggesting it may reduce AF risk.

In fact, among the PWH, more patients with AF than controls were found to be not using ART (16% vs. 12%). Not being on the ART was associated with an OR of 2.86 (95% CI: 1.39–5.88) [72].

AF treatment in the PWH remains challenging as compared with the control group, the PWH have higher inpatient mortality rates (9.2% vs. 5.1%) and longer hospital stays. One hindrance might be the interactions between ART and cardiovascular drugs. For example, among anti-arrhythmics, only sotalol can be used in standard doses with all ART drugs [73].

Additionally, recurrence rates 5 years following catheter ablation were significantly higher in the PWH (100% vs. 54%, *p* < 0.001) [74], with the two most common arrhythmias during the follow-up period being atrial flutter (61.3%) and AF (29%). During an isoproterenol challenge in patients with repeat ablation, non-pulmonary vein triggers were more prevalent among the PWH (93.5% vs. 54%, *p* < 0.001). However, there were no significant differences between the groups 1 year after repeat ablation (*p* = 0.753), suggesting that a different approach to ablation in the PWH could yield better outcomes.

Similarly to HIV, chronic hepatitis C (HCV) infections have been associated with an increased risk of AF. Patients with HCV who received antiviral treatment had lower incidental AF compared to infected controls without treatment [75]. Furthermore, HCV infection was associated with a higher incidence of atrial fibrillation than chronic hepatitis B (HBV) infection (HR 1.25, *p* = 0.070) [76].

For both HIV and HCV, there is evidence of chronic inflammation [77,78,79,80] that could potentially be related to the onset and prognosis of AF in infected patients. This is supported by findings that non-alcoholic fatty liver disease and alcohol consumption are individually linked to AF risk [81,82,83], as both can activate pathways promoting inflammation.

### 2.5. Autoimmunity and Its Relation to Atrial Fibrillation

Several studies concerning the possible relationship between autoimmune diseases and AF were conducted. Tilly et al. [84] found out, through the analysis of data from the UK Biobank, that there is an increased chance of developing new-onset AF in patients with rheumatic fever, Crohn’s disease, ulcerative colitis, rheumatoid arthritis, polyarteritis nodosa, systemic lupus erythematosus, or systemic sclerosis. Notably, among these conditions, ulcerative colitis was the only one associated with a stronger likelihood of new-onset AF in men, while the other diseases were linked to a higher risk in women.

A similar study specifically focused on rheumatic diseases. Data analysis by Khan et al. [85], showed a higher odds ratio of AF in patients with gout (OR = 1.25), vasculitis (OR = 1.19), polymyalgia rheumatica (OR = 1.15), dermatopolymyositis (OR = 1.14), psoriatic arthropathy (OR = 1.12), lupus (OR = 1.09), rheumatoid arthritis (OR = 1.05), and pseudogout (OR = 1.04). Additionally, they observed the highest prevalence of AF in patients with polymyalgia rheumatica (33.2%), followed by patients with gout (30.2%) and pseudogout (27.1%). Although the exact cause of this phenomenon is not fully explained, it is worth noting that the study population with polymyalgia rheumatica had the highest mean age among rheumatic diseases, which is one of the main risk factors for AF.

Other studies also support an association with other rheumatological diseases and an increased risk of this arrhythmia, with HRs of 2.84 and 1.39, respectively for systemic lupus erythematosus and rheumatoid arthritis [86]. Moreover, patients with untreated inflammatory bowel diseases displayed changes in left atrial morphology, including increased left atrial volume, P-wave dispersion, and atrial electromechanical delay [87]. Research by Kristensen SL et al. [88], in a 24,499-person group with inflammatory bowel disease, showed a two times higher risk of AF and a 1.5-fold higher risk of stroke during active stages, compared to patients in remission.

It is also important to consider that medications used in therapeutic immunotherapy may be an additional risk factor. Potent anti-inflammatory drugs may cause the development of AF through different mechanisms. Furthermore, autoimmune diseases may lead to lifestyle changes, such as less physical activity due to joint pain.

As shown above, there is a clear association between inflammatory diseases, especially autoimmune diseases, and atrial fibrillation, although the mechanism of this association remains partly unclear. Future research should explore the impact of medications, lifestyle changes, and other potential risk factors to understand better how inflammatory processes contribute to the development of AF.

### 2.6. Hormonal Imbalance as a Pro-Inflammatory Risk Factor for Atrial Fibrillation

The most common thyroid dysfunctions are related to autoimmunity and heightened inflammatory activity in the thyroid gland. Hypothyroidism is most frequently associated with Hashimoto’s disease (HD). This autoimmune condition leads to thyroid dysfunction, while hyperthyroidism is predominantly linked to Graves’ disease (GD), characterized by excessive thyroid gland activation driven by autoimmunity. Both conditions are recognized as risk factors for CVD, including CAD, hypertension, atherosclerosis, and CVD-related mortality [89,90,91].

HD and GD are both associated with elevated levels of pro-inflammatory cytokines, such as IL-1β, IL-6, IL-8, IL-10, IL-12, and TNF-α [92,93,94] in the bloodstream, contributing to a pro-inflammatory state. HD, like atrial fibrillation (AF), involves NF-κB activation, which, in turn, drives the activation of the NLRP3 inflammasome [95]. Additionally, hypothyroidism has been linked to increased cardiac fibrosis through upregulation of α-SMA expression, promoting the differentiation of fibroblasts into myofibroblasts [96].

Hypothyroidism may also indirectly contribute to AF risk by worsening obesity and increasing the risk of obesity-correlated AF risk factors such as hypertension and CAD. In hyperthyroidism, elevated levels of free triiodothyronine (fT3) and free thyroxine (fT4) increase sympathetic activity, altering myocardial electrophysiology and further shortening action potential duration, as well as increasing the likelihood of spontaneous activity in pulmonary vein cardiomyocytes and after-depolarizations in atrial cardiomyocytes [97].

Studies in animal models have shown that [98] both hypo- and hyperthyroidism can affect myocardial electrophysiology, potentially raising susceptibility to AF. However, the relationship between thyroid dysfunction and AF risk is less evident in humans. While hyperthyroidism has been conclusively associated with a heightened risk of AF, the link between hypothyroidism and AF remains uncertain. For example, in a Danish cohort [99], hypothyroidism was associated with a decreased risk of AF onset. A meta-analysis of 11 cohorts by Baumgartner et al. [100] also failed to prove a positive correlation between AF and hypothyroidism. Nevertheless, the recent meta-analysis by Singh et al. [101] did show that hypothyroidism might be associated with an increased risk of AF. However, due to the limitations of observational studies and meta-analyses based on observational studies, further research is necessary to definitively clarify the relationship between hypothyroidism and AF risk.

Another hormone that has been reported to alter the risk of CVD is melatonin, which, although primarily associated with regulating the sleep–wake cycle, has been widely reported to pose antioxidative and anti-inflammatory properties [102,103,104,105]. Nevertheless, melatonin’s role in the management of AF is still under evaluation. In an animal model by Qin et al., melatonin potentially increased the risk of AF among obese rats via increased lipid stress and impairment of atrial Akt signaling [106]. Moreover, while melatonin supplementation did not reduce the risk of POAF, it was noted to decrease inflammation markers and duration of AF [107,108].

## 3. Nonsteroidal Anti-Inflammatory Drugs

Nonsteroidal anti-inflammatory drugs (NSAIDs) are currently among the most widely used medications; they are mainly prescribed as analgesics and anti-inflammatory treatments due to their shared mechanism of action involving the inhibition of cyclooxygenase (COX) enzymes, which results in reducing the production of prostaglandins (PGs) and, in turn, inflammatory agents [109]. Peculiarly, despite the straightforward primary pharmacodynamic property, it has been hypothesized that by intermittent downregulation of inflammatory pathways, NSAIDs may lead to a compensatory upregulation, shifting the balance to an inflammatory state [110]. This might explain their role in the exacerbation of multiple cardiological conditions, including progression from paroxysmal to persistent AF [111] and risk of new AF onset.

However, the exact mechanism and consistency among different NSAIDs needs further research.

Before the establishment of their connection with cardiovascular events, COX-2 selective inhibitors (coxibs) were regarded as a significant therapeutic opportunity for patients with CVDs. After the withdrawal of rofecoxib in 2004, the risk linked to coxib use was thoroughly examined. According to a then-published Swedish cohort of 7 million subjects [112], etoricoxib was associated with incident AF (HR = 1.35; 95% CI 1.19–1.54). However, celecoxib was not. Nevertheless, celecoxib was shown to prolong P-wave duration in electrocardiograms of inflammatory arthritis patients [113], which may suggest arrhythmia predisposition. Thus, this group of therapeutics should be restricted to patients who could not have benefitted from other substances.

A meta-analysis of eight available publications by Chokesuwattanaskul et al. [114] confirmed previous assumptions that some non-selective NSAID usage was associated with a significantly increased risk of AF (RR = 1.29; 95% CI 1.19–1.39, I^2^ = 68%). Notably, the result was consistent across three analyzed NSAIDs (Diclofenac, Ibuprofen, and Naproxen).

The mechanism explaining this relation has yet to be fully understood. Nevertheless, one possible solution may be related to the inhibition of COX enzymes found in the kidney [115]. Prostaglandins produced in the kidney play a crucial role in compensatory mechanisms when renal perfusion is decreased [116]. Thus, inhibition of their synthesis by the NSAIDs causes fluid retention and, in turn, increased blood pressure, leading to a higher risk of AF onset [117].

In particular, aspirin, which is a potent antiplatelet agent, has historically been associated with a lower risk of thrombus formation [118] in the left atrial appendage; however, the most recent guidelines provided by AHA/ACC/HRS 2023 [119] and ESC/EHRA 2024 [2] do not recommend a single antiplatelet treatment over VKA or NOAC administration due to inferiority in stroke prevention. NSAIDs, both non-selective and COX-2-selective, are also known to increase the risk of bleeding in patients taking NOACs and VKAs, which is the rationale for their discouragement among patients with AF.

## 4. COVID-19 Treatment and Targeted Drugs

An outburst of the SARS-CoV-2 2019 (COVID-19) pandemic has accelerated development in various medical fields, including reshaping our understanding of AF. Cardiac implications of the COVID-19 infection have been researched since the virus’s discovery. However, their exact impact on patients’ prognoses and appropriate therapeutic approach remained unclear. A meta-analysis by Romiti et al. [120] found that 8% of COVID-19 patients had comorbid AF. Moreover, both diseases were proven to share some underlying inflammatory markers such as IL-6 and C-reactive protein [121].

COVID-19 infection is characterized by an intense reaction from the immune system, known as the cytokine storm [122], which rationalizes utilizing biological treatment, including anti-IL-6 monoclonal antibody, tocilizumab [123]. Multiple studies on its administration among COVID-positive patients were conducted during the past years, few of which have examined its potential role in atrial fibrillation onset and management. Patel et al. [124] established an analysis of all systematic reviews regarding the efficacy and safety of tocilizumab and found no significant differences in the new onset of AF (*p* > 0.05). Nevertheless, the most plausible mechanism in which tocilizumab could help manage the risk of AF onset is its potential to reduce the QTc interval duration [125].

Chloroquine (CQ), an antimalarial drug, has been identified with in vitro antiviral properties for over 50 years [126]. However, most clinical trials have failed to show any benefit from such prescription in severe viral infections. Thus, with the new pandemic, multiple researchers have started investigating whether it could help treat SARS-CoV-2 infection. However, none of them proved it to be effective in patients with severe COVID-19 [127]. Nevertheless, growth in drug admission has led to multiple exciting discoveries. One is the establishment of corrected QT (QTc) prolongation due to CQ admission [128]. The study found that treatment with CQ has led to a mean QTc prolongation of 35 ms (95% CI 28–43 ms) in COVID-19 patients. The results were most likely associated with the blockade of the rapidly activating delayed rectifier K^+^ current, previously reported in patients prescribed antimalarial drugs [129]. Furthermore, CQ has been established to prolong an action potential duration, which has led to a cease of arrhythmia in the mechanism of re-entrant excitation on a 3D human atria model [130]. Similarly, CQ was effective in terminating stretched-induced AF in an isolated sheep’s heart [131]. Before the restoration of sinus rhythm, CQ re-entry frequency was shown to be reduced from 10.6 ± 0.7 Hz in control to 6.3 ± 0.7 Hz (*p* < 0.005). Moreover, CQ increases the rotor core size, and in AF, it tends to be smaller than sinus rhythms [132]. Considering the aforementioned, CQ can potentially terminate persistent AF. However, clinical trials are needed to exhibit its usefulness.

Another medication that was expected to be helpful in COVID-19 management is hydroxychloroquine (HCQ), a less toxic derivative of CQ. Similarly to CQ, HCQ was primarily used in malaria management; however, over the years, it has been proven to be a potent anti-inflammatory agent against many autoimmune diseases [133]. Most enthusiasm regarding its’ potential in treating patients with SARS-CoV-2 came from in vitro studies, which suggested that it could have similar antiviral effects as CQ [134]. However, the initial optimism was not long-lasting, and HCQ has yet to be proven effective in this group of patients [127]. Nevertheless, in the case of AF, HCQ usability seems more apparent. Firstly, HCQ use was associated with decreased incident AF in patients with Systemic Lupus Erythematosus (OR = 0.12, 95% CI: 0.034–0.39, *p* = 0.0005) [135]. Given the additional burden to cardiovascular health caused by autoimmune diseases, this should be researched in prospective studies.

Cytokine storm can potentially be managed with the use of naturally obtained compounds e.g., quercetin was shown to limit pro-inflammatory cytokines rise in lipopolysaccharide-induced mice [136]. Quercetin is commonly found in various edible plants [137]. It was established to have multiple antioxidant mechanisms including inhibition of lipid peroxidation. Its usefulness against AF is most likely connected to its ability to modulate the MAPK signaling pathway [138]. This property may explain its potential to limit atrial remodeling [139,140] and, in turn, ameliorate atrial fibrillation; however, more research needs to be established to fully explore quercetin’s potential.

## 5. Targeted Anti-Il-1B Treatment

Il-1β (interleukin 1β) plays a pivotal role as a byproduct of NLRP3 activation, which is involved in paracrine signaling of the inflammatory cascade, thus promoting local inflammation. Moreover, researchers have established an IL-1β connection to pressure overload-induced AF in mice. To our current knowledge, only one significant attempt was made to evaluate the potential role of Il-1β blockade in managing AF using canakinumab—an anti-IL-1β antibody primarily approved for treating cryopyrin-associated periodic syndrome, Muckle–Wells syndrome, and familial cold auto-inflammatory syndrome, in a randomized, double-blinded trial, CONVERT-AF [141] (Canakinumab for the Prevention of Recurrences After Electrical Cardioversion). This ended with the authors stating that there was no significant reduction in AF recurrences after cardioversion in patients with persistent AF.

## 6. Glucocorticosteroids

Glucocorticosteroids, also known as steroids, are a group of derivatives of cortisol—an endogenous hormone secreted from adrenal glands, primarily associated with its role in metabolism and potent anti-inflammatory properties—and are currently being utilized in the management of a plethora of diseases, mainly linked to the dysregulation of the immune system such as autoimmune disorders or hematological malignancies. The mechanism by which steroids are linked to the pathogenesis of AF is complex, as the pharmacodynamic properties of said drugs have not yet been fully elucidated despite over 70 years since their first clinical introduction. The most commonly accepted theory involves deactivating inflammatory genes by reversing histone acetylation (inhibition of histone acetyltransferase) and switching on anti-inflammatory genes. The complex translocates to the nucleus when steroids bind to their receptors in the cytoplasm. In the nucleus, homodimers bind to glucocorticoid response elements (GREs) and GRIP-1, pCAF, or CBP, resulting in the synthesis of anti-inflammatory particles, GILZ, SLPI, MKP-1, and IκB-α [142].

Regrettably, the use of steroids is associated with side effects, including increased cardiovascular risk [143], which stems from the fact that steroids can bind with glucocorticoid receptors (GRs) and mineralocorticoid receptors (MRs) in cardiomyocytes [144]. High doses of steroids downregulate the expression of GRs and upregulate the expression of MRs, resulting in increased binding with the latter receptors [144]. Activation of MRs promotes inflammation and leads to cardiac fibrosis [145,146]. Moreover, the relation between NLRP3 inflammasome and MR activation has also been established [147].

Cruz-Topete et al. [148] argue that this paradoxical outcome’s function is stimulating a response to a stressor and returning to homeostasis afterwards. Even though the mechanism behind those contradictory properties is not yet fully known, there are certain factors, which, apart from the aforementioned, might play a role in a pro-inflammatory response. One of those is steroids’ ability to stimulate the expression of TLRs, such as TLR2 and TLR4, which are also linked to the activation of the NLRP3 inflammasome. Moreover, cases of glucocorticoid-induced atrial fibrillation have been reported, mainly after the administration of high doses; however, further research in this matter is needed [149].

Nevertheless, the anti-inflammatory properties, e.g., cardioprotective properties of the heat shock [150] protein release stimulated by the corticosteroids, could have utility in certain clinical settings, such as prevention of POAF. In a 2017 study, patients undergoing elective coronary artery bypass grafting were given 1 g of methylprednisolone before the procedure and 100 mg of hydrocortisone every 8 h for 3 days after surgery, which lowered the incidence of postoperative AF [151]. Another study showed that administering methylprednisone before surgery and dexamethasone after reduced IL-6 levels and was associated with reduced cases of postoperative AF onset [152].

The latest meta-analysis included 14,442 patients and revealed that low-dose corticosteroids seem to reduce the incidence of POAF (RR 0.81, 95% CI 0.71–0.92, *p* = 0.001) in a non-dose-dependent manner [153].

However, a larger meta-analysis (16,013 randomized patients) revealed that only small trials showed reduced incidence of POAF resulting from steroids. It is also important to note that myocardial injury happened more often in patients who were given steroids, and there was no statistically significant difference in mortality between the steroid and placebo group (RR 0.85; 95% CI 0.71–1.01; *p* = 0.07; I^2^ = 0%) [154].

There have also been attempts to use steroids to prevent the recurrence of atrial fibrillation after catheter ablation; a 2017 study found that oral corticosteroids given prior to ablation did not impact the incidence of postoperative AF [155].

A single bolus of steroids, irrespectively of dose (low or moderate), also did not prevent atrial fibrillation recurrence after radiofrequency catheter ablation, even though patients who received a moderate dose of methylprednisolone (125 mg) had significantly lower markers of inflammation [156].

A 2018 meta-analysis concluded that corticosteroids are effective only in preventing early recurrence of AF after ablation [157]. According to another study, preoperative steroids do not lower the risk of AF after pneumonectomy [158]. Although it seems that corticosteroids play a role in the prevention of postoperative AF after heart surgeries, there is no evidence of similar effects in different situations. Moreover, a risk–benefit ratio does not rationalize the utilization of steroids in the prevention of POAF, especially since high-dose steroids exert a plethora of side effects [159].

## 7. Vitamin C

Ascorbic acid (vitamin C), as well as its antioxidative properties, is also believed to have an anti-inflammatory effect by interfering with the NFκB/TNFα pathway, resulting in decreased levels of pro-inflammatory cytokines [160]. Considering that fact, there have been many attempts to use ascorbic acid in the prevention of atrial fibrillation after cardiac surgeries. According to a phase 1 pilot study of 20 patients undergoing AF ablation, high-dose intravenous ascorbic acid administered before the procedure minimized the spike of postoperative CRP levels. However, no impact on early reoccurrence of AF was observed [155]. A 2014 study of 100 patients who received a coronary artery bypass grafting surgery showed a reduced incidence of postoperative atrial fibrillation in patients who were given oral vitamin C before surgery and for 5 days after (32% in the control group vs. 8% in the treatment group, *p* = 0.003).

Moreover, there was a significantly lower length of hospital stay in the treatment group [161]. Nevertheless, a larger-scale study of 314 patients undergoing CABG did not present superiority of the vitamin C administered intravenously pre- and postoperatively combined with oral vitamin C after the procedure, as it did not affect either AF occurrence or mean hospitalization time [162]. Ascorbic acid has not proven its role in preventing POAF among CABG patients (control vs. study, 18.9% and 13.5%, respectively; *p* = 0.314 [163]). To date, the administration of ascorbic acid and its role in preventing AF remains elusive.

## 8. Colchicine

Colchicine is an anti-inflammatory drug, commonly administered as prophylaxis and in treating gout flares and managing pericarditis [164,165]. Moreover, due to its potent anti-inflammatory properties, colchicine has also been widely acknowledged as an effective drug in the treatment of Familial Mediterranean Fever (FMF), Behçet’s syndrome, pericarditis, and postcardiotomy syndrome [166]. At high doses (100 ng/mL or higher) colchicine affects gene expression. Short-time exposure (30–120 min) causes changes in genes involved in the cell cycle and its regulation, while long-time exposure (12–24 h) alters neutrophil migration or other inflammatory processes [167]. By disrupting microtubules, colchicine inhibits ROS production, which is crucial for inflammasome activation [168,169]. Furthermore, colchicine inhibits the release of histamine from mast cells, mitigates myocardial fibrosis, and induces the hormone GDF-15 in the liver, which suppresses myeloid cell activation [170,171,172,173]. However, the most important target of colchicine’s anti-inflammatory properties is the active suppression of the release of IL-1-β and IL-18 through direct inhibition of the NLRP3 inflammasome [174,175], which attracted significant attention, especially in the field of cardiology.

The potential role of colchicine in the prevention of ischemic cardiovascular events has been speculated and proven in patients with a recent myocardial infarction (MI). Patients exposed to colchicine at a dose of 0.5 mg daily were less likely to succumb to cardiovascular causes, resuscitated cardiac arrest, myocardial infarction, stroke, or urgent hospitalization from angina leading to coronary revascularization [176].

Additionally, numerous studies have demonstrated that colchicine is an efficient prophylaxis for POAF. The effects are most probably connected to its anti-inflammatory properties, while inflammation marker levels are associated with a higher incidence of POAF [177,178]. A meta-analysis of RCTs concluded that colchicine distinctly lowers the occurrence of POAF [179] (incidence of POAF in studied population vs. control 18.2% vs. 26.8%, *p* < 0.01, n = 1885); however, as mentioned by the authors, colchicine administration was linked to an increased risk of gastrointestinal adverse effects [179] including nausea, vomiting, or diarrhea.

Another meta-analysis of RCTs found that colchicine decreases AF recurrence after pulmonary vein ablation and in post-cardiac surgery patients [177]. Other meta-analyses and systematic reviews emphasized the advantages of colchicine in post-cardiac surgery patients but acknowledged no substantial benefit in patients with coronary artery disease [180]. Further studies are required to identify the optimal dosage and length of colchicine therapy needed to minimize side effects and enhance its efficacy in preventing AF [181].

While colchicine provides substantial therapeutic benefits, its narrow therapeutic window creates a threat for major side effects, particularly in cases of overuse. Typical adverse effects include gastrointestinal issues such as nausea, vomiting, and diarrhea, observed in 5–10% of patients [182]. Rare but severe side effects, such as myopathy, neuropathy, bone marrow suppression, and acute pancreatitis, have been documented, particularly in patients with renal dysfunction or during polypharmacy [182]. Moreover, colchicine’s pharmacokinetics include metabolism through the gastrointestinal tract, with important roles played by P-glycoprotein and CYP3A4 enzymes, raising the likelihood of drug interactions [183,184,185].

## 9. Statins

The therapeutic role of statins involves a multifaceted process that begins with establishing a low-cholesterol environment within the cytosol of hepatocytes, with reversible competitive inhibition of HMG-CoA reductase. An induced low-cholesterol state triggers a series of events, including the upregulation of transcription of the LDLR gene. This leads to an elevation in the number of LDL receptors on the cellular membrane, facilitating more efficient uptake of the LDL-C particles from the blood serum. However, the therapeutic effect of statins extends beyond their impact on cholesterol synthesis and LDL receptor expression, as statins have been widely accepted to possess potent anti-inflammatory properties. The initial efforts to establish the correlation between statin therapy and decreased systemic inflammation were undertaken by M. A. Albert et al. [186] through the conduction of the PRINCE study (The Pravastatin Inflammation CRP Evaluation trial). This resulted in the demonstration of a significant reduction in hs-CRP levels after 24 weeks of pravastatin therapy compared to the placebo group (mean 37.8% reduction, *p* = 0.002).

Mechanism of the anti-inflammatory properties of the statins can be attributed to, for example, impairment of the mevalonate pathway as it delivers intermediates for the synthesis of various compounds, such as geranylgeranyl pyrophosphate (GGPP) and farnesyl pyrophosphate (FPP), which are essential substrates for the synthesis of signal proteins involved in numerous intracellular pathways [187]. Such pathways include isoprenoids and subsequent isoprenylation of small proteins—critical components of interleukin synthesis (e.g., Il-6, TNF-α, IL-1b) [188], ROS generation, and cellular proliferation [189]. Additionally, statins have been found to activate the sphingosine-1-phosphate pathway, known for exerting anti-inflammatory and anti-atherothrombotic effects through increasing endothelial NO production and response [190].

**Figure 3:**
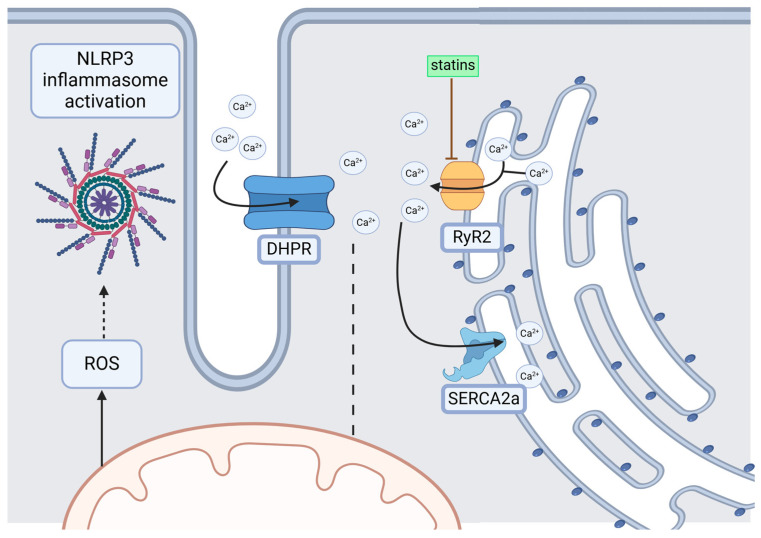
The role of overactivation of cardiac type 2 ryanodine receptors in the promotion of inflammation via increased ROS generation and subsequent NLRP3 inflammasome activation. Adapted from Xu et al. [200]. RyR2—ryanodine receptor 2, SERCA—Sarcoendoplasmic Reticulum Calcium ATPase, DHPR—dihydropyridine receptor, ROS—reactive oxygen species. Created with BioRender.com.

Regarding the relationship between statin anti-inflammatory properties and AF pathogenesis, it is well acknowledged that statins can attenuate the activity of the NLRP3 inflammasome [191], a key player in the electrical remodeling of the atrial myocardium. Moreover, statin therapy efficiently regulates TGF-β-related pathways, which are also associated with promoting a profibrotic environment [192,193,194,195,196]. More recently, another pathway has been proposed, related to fibrosis of the atrial myocardium and AF pathogenesis, involving reduced uptake of Ca^2+^ ions from the cytosol by the mitochondria in a profibrotic environment, which increases Ca^2+^ concentration and further promotes Ca^2+^ signaling pathways involving pathological overactivation of cardiac type 2 ryanodine receptors (RyR2) and increased oxidative stress, which fuels NLRP3 inflammasome activation [Figure 3]. Several in vitro studies have also found potential mechanisms of statin anti-arrhythmic properties involving attenuating L-type Ca^2+^ current and oxidative stress on the myocardium, induced by angiotensin II through inhibiting NOX2/gp91phox and P47phox synthesis [197,198,199]. Simvastatin has also been associated with reducing the open probability of cRyR2, which might also exert protection against Ca^2+^ ion leaks [200] and, consequently, Ca^2+^-dependent arrhythmias and oxidative stress. Another potential mechanism underlying the beneficial role of statin therapy as an upstream therapy has also been proposed by Yeh YH et al. [201] involving the activation of the Akt/Nrf2/HO-1 pathway, which delivers heme oxygenase-1 (HO-1)—a potent antioxidant that mediates the suppressive effect of statins on atrial tachycardia-induced structural remodeling in an in vitro model.

Outside of the scope of the anti-inflammatory properties of statin therapy, other pleiotropic effects also play a protective role against AF, as proposed by Andelova et al. [202]. Statin-induced modifications of the lipid raft [203,204], which function as a frame for ion channel regulatory proteins, might also influence the conductance of the ion channels. Other potential beneficial effects of statin administration and arrhythmia risk have been correlated with Cx43 (connexin 43)-related pathways involving pathological activation of the RAAS (renin–angiotensin–aldosterone system) [205,206], PI3-kinase/Akt pathway activation [207], and stabilization of acetylcholine-activated K^+^ current [208].

The benefits of statin administration as an upstream therapy [209] for preventing AF and reducing AF-related complications have been widely recognized in several real-life trials and population-based studies. In a meta-analysis of 100,287 patients, by Pastori et al. [210], statin administration among patients with AF was correlated with a reduction in all-cause and cardiovascular mortality by 41% and 25%, respectively. A study by Huang JY. et al. [211], on a group of 52,490 patients with newly diagnosed AF, provides convincing evidence of statin therapy as a preventive measure against the incidence of HF, as statin use was associated with a 19% lower risk of HF (HR 0.81 [95% CI, 0.78–0.85]). Statin therapy has also been found to decrease the risk of AF onset after cardiac surgery during the secondary analysis of the SEARCH-AF trial [212], as patients treated with statins at discharge had a twofold lower rate of POAF (postoperative atrial fibrillation) than those without prescribed statins (18.4% vs. 8.1%, log-rank P ¼ 0.0076). Researchers also suggest that the effect is probably dose-dependent as more intensive statin therapy yielded better results (log-rank P trend ¼ 0.0082). Statins have also been proven to reduce the risk of ischemic stroke among AF patients [213,214,215], providing reassurance and confidence in their potential benefits. Furthermore, according to Uchida et al. [216], statin therapy is associated with a lower risk of major bleeding events among patients undergoing NOAC treatment (HR = 0.77 [0.63–0.94]), which adds potentially another layer of the benefit of statin therapy among AF patients who undergo polypharmacotherapy or have estimated high-risk of major bleeding due to comorbidities.

## 10. Omega-3 Polyunsaturated Fatty Acids

Omega-3 pertains to a group of polyunsaturated fatty acids (PUAFs) with the double bond found at the third carbon from the methyl end of the fatty chain. The most crucial members of the omega-3 fatty acid family, in terms of their importance in physiology, are EPA (eicosapentaenoic acid) and DHA (docosahexaenoic acid) [217]. Since we do not possess the ability to synthesize endogenous omega-3 fatty acids, proper supplementation of it is paramount which has been acknowledged by the AHA [218] and ESC guidelines [219] due to their widely reported role in reducing triglyceride levels in blood serum [220,221,222], decreasing blood pressure [223,224], and their potent anti-inflammatory properties [225,226,227] [228].

The initial correlation between potential anti-inflammatory properties and cardiovascular benefits of PUAFs dates back to the 1980s, as researchers at the time sought to connect the reduction in prostaglandin (namely thromboxane A2) synthesis, which resulted in antithrombotic and cardioprotective effects, with the potent PUAFs intake observed among the Inuit population. Currently, we know that EPA and DHA’s role in homeostasis goes beyond reducing thromboxane A2 activity, as a plethora of studies have found additional mechanisms of action for omega-3 fatty acids, including antioxidative properties. In particular, DHA, which results from its presence in the mitochondrial membrane and role in mitochondrial pathways [229]. Moreover, PUAFs have been reported to decrease substantially pro-inflammatory cytokines such as Il-6 and TNF-α [230] and downregulate nuclear factor kappa B (NF-κB), an essential mediator of the NLRP3 inflammasome [231]. PUAFs are also directly linked to the attenuation of mechanisms of AF pathophysiology, including the regulatory function of Ca^2+^ ion distribution within the cardiomyocytes, alteration in the cellular membrane and ion transporters, sympathetic–vagal homeostasis, and cardioprotection, through the upregulation of Cx43 [202,232].

Uncertainty regarding the effectiveness of omega-3 fatty acids in managing AF remains despite their proven role in managing hypertriglyceridemia and anti-inflammatory properties. Huh, JH et al.’s [233] review of large trials involving PUAF administration, and their potential benefits in managing cardiovascular diseases, noted a lack of correlation between omega-3 intake and reduced AF onset risk. Additionally, the REDUCE-IT [234] and STRENGTH [235] trials, involving high-dose omega-3 fatty acids, showed a significant increase (*p* = 0.003) in AF risk among the studied population by 5.3% and 3.9%, respectively. Results regarding the role of PUAF administration in reducing POAF incidents were also mixed, as Driscoll et al. [236] reported. The mechanisms of PUAFs’ potential role in AF pathogenesis remain unclear. However, hypotheses include their regulation of PIEZO ½ ion channel activity and omega-3 fatty acids’ reported role in increasing vagal tone [237].

## 11. Vitamin D

Vitamin D is a group of compounds classified as secosteroids, commonly associated with their role in calcium–phosphate metabolism and potent immunomodulatory properties related to the attenuation of IFN-γ, TNF-α, IL-1β, IL-6, IL-8, IL-12, and IL-17 secretion, and increased anti-inflammatory cytokines IL-4 and IL-10. Moreover, vitamin D has been proven to regulate the immune system directly via VDRs (vitamin D receptors) on leukocytes, which additionally promotes Treg activity [238] and suppresses Th17 cytokine production [239]. A relation between vitamin D and RAAS has also been established as studies have shown that vitamin D deficiency increases renin levels [240,241] and thus might also increase the risk of hypertension—one of the major risk factors of atrial fibrillation [242]. Regarding one of the major drivers of atrial fibrillation—the overactivation of NLRP3 and its relationship with vitamin D—several studies have reported that vitamin D decreases NLRP3 gene expression and its activation through impeding BRCC3-mMediated deubiquitination of NLRP3 [243]., thus inhibiting further excretion of profibrotic cytokines IL-1β and IL-18 [244,245,246].

In a meta-analysis of five randomized controlled trials conducted by Ding et al. [247], a deficiency in vitamin D, defined as levels below 20 ng/mL, was associated with an increased risk of atrial fibrillation (HR: 1.12, 95% CI: 1.005–1.25). However, there is reassurance in the form of vitamin D supplementation. For every 10 ng/mL increase in vitamin D level, the risk of atrial fibrillation decreased by 5% (HR: 0.95, 95% CI: 0.93–0.97). In a large RCT based on the Finnish population [248], patients who underwent the supplementation of 1600–3200 IU/d had a significantly lower chance of developing AF compared to the placebo group (*p* = 0.02). In a meta-analysis of three RCTs by Ansari et al. [249], it was found that preoperative vitamin D supplementation in patients undergoing CABG significantly reduced the risk of POAF onset (RR = 0.6, 95% CI = 0.4–0.9, *p* = 0.01), providing confidence in the potential of vitamin D to improve patient outcomes.

Despite the promising results, the limitations of RCTs involving vitamin D and the risk of atrial fibrillation onset underscore the need for further studies.

## 12. Antidiabetic Drugs and Atrial Fibrillation

Research has illustrated that various classes of antidiabetic drugs can affect the incidence of AF differently [250]. Medications used in managing diabetes mellitus, such as sodium–glucose cotransporter-2 (SGLT2) inhibitors, are acknowledged for their anti-inflammatory and antifibrotic properties. It has been hypothesized that SGLT2 inhibitors indirectly affect AMP-activated protein kinase (AMPK) to reduce mTOR pathway activity, which is known to regulate inflammatory responses in macrophages and dendritic cells [251,252]. Moreover, SGLT2 inhibitors decrease pro-inflammatory cytokines IL-6, IL-1beta, and TNF-alpha production and reduce the M1/M2 macrophage ratio [253]. SGLT2 inhibitors also impact other inflammatory tracts such as the NLRP3 inflammasome, expression of Toll-like receptor 4 (TLR-4), and activation of nuclear factor kappa B (NF-κB) [254]. By affecting inflammation, oxidative stress, the renin–angiotensin system, and metabolic activities, SGLT2 inhibitors can prevent lung, heart, liver, and kidney fibrosis [252]. These characteristics assist in decreasing the risk of AF and provide cardioprotective effects [255,256,257].

Recent studies have underscored the potential of SGLT2 inhibitors in preventing AF. The DECLARE-TIMI 58 study, a significant milestone in the research on SGLT2 inhibitors, demonstrated the potential of dapagliflozin in preventing AF. This study, involving 17,160 patients with diabetes mellitus (DM), revealed that dapagliflozin reduced the risk of AF and atrial flutter (AFL) events by a substantial 19% [258]. These findings were further validated by a systematic review and meta-analysis of 16 randomized controlled trials, which included 38,335 patients with type 2 DM. The meta-analysis found a significant reduction in AF/AFL and all-cause mortality in individuals treated with SGLT2 inhibitors [259]. Another systematic review and meta-analysis of 22 randomized controlled trials also supported these findings, showing that SGLT2 inhibitor use in patients with DM, chronic kidney disease (CKD), or (HF) was associated with a lower incidence of AF and embolic stroke [260]. A retrospective study, including 326 patients with type 2 DM and AF, who underwent radiofrequency catheter ablation (RFCA), further reinforced these results. The dapagliflozin group (n = 79) had an almost two times lower recurrence rate than the control group (n = 247) (27.8% vs. 44.9%, *p* = 0.007). The mean follow-up in this study was 15.5 ± 8.9 months [261].

Another SGLT2 inhibitor is empagliflozin, which has also been studied extensively, including the EMPEROR-Preserved trial, which involved 5988 patients. Although the study showed no effect on new-onset AF [8% in empagliflozin and 8.1% in placebo-developed AF; HR 1.00 [95% CI 0.77, 1.29], (*p* = 0.98)], empagliflozin reduced the risk of severe HF events and slowed the eGFR decline in patients with HF and an ejection fraction above 40%, regardless of baseline AF [262]. Other studies supported that empagliflozin had less impact on AF risk than dapagliflozin [263]. However, both studies pointed out their limitations related to the short follow-up. Moreover, these results are not confirmed by the meta-analysis of six RCT, including 9467 patients, which revealed that empagliflozin resulted in a significant and higher reduction in the risk of new-onset and recurrent events of AF (RR 0.55, 95% CI 0.34–0.89; *p* = 0.01) in comparison to dapagliflozin (RR 0.69, 95% CI 0.43–1.11; *p* = 0.12) [264]. Also, in the study of Engstrom, which consists of 79,343 patients with newly added SGLT2 inhibitors, a significant reduction in new-onset AF development was noted, particularly in empagliflozin users [265]. This effect can be explained by the reduction in overall Na influx in cardiomyocytes caused by empagliflozin, which is linked to a lower risk of arrhythmias [266].

The effectiveness of other diabetic medications in preventing AF can vary. A meta-analysis involving 130,854 diabetic patients found that those treated with thiazolidinediones (TZDs) had an approximately 30% lower risk of developing AF compared to controls. Notably, this reduction was statistically significant with pioglitazone use but not with rosiglitazone [267]. Another study, which included 645,710 patients with type 2 diabetes, showed that metformin also offered protection against new-onset AF [250]. Interestingly, the combination of metformin and TZDs proved more effective in preventing AF than either drug alone [250].

Furthermore, glucagon-like peptide-1 receptor agonists (GLP-1RAs) were significantly more effective than other diabetes medications, such as metformin, sulfonylureas, and insulin, in reducing AF/AFL events. Patients using dipeptidyl peptidase-4 (DPP-4) inhibitors as a second-line therapy after metformin had a lower risk of new-onset AF compared to those using non-DPP-4 diabetes drugs as a second-line treatment [268]. Another study demonstrated that users of biguanides or TZDs had a reduced risk of developing new-onset AF compared to those who did not use diabetes medications. In contrast, insulin users were more likely to develop AF compared to non-users [269].

Metformin, a widely used antidiabetic drug, has been shown to reduce metabolic burden and inflammation, potentially leading to a decrease in atrial fibrosis and AF [270]. It also mitigates rapid pacing-triggered atrial myocyte structural alterations by reducing inner cell distress. Furthermore, metformin addresses impaired insulin sensitivity associated with a lower AF vulnerability [270].

Thiazolidinediones (TZDs) have different mechanisms of action. They function as peroxisome proliferator-activated receptor gamma (PPARγ) activators, which enhance insulin activity, reduce impaired insulin sensitivity, and modulate compounds influencing insulin function and lipid processing [270,271,272]. TZDs also have antioxidant and anti-inflammatory effects, probably by regulating signal transduction pathways engaging tumor necrosis factor-α (TNF-α), transforming growth factor-β (TGF-β), superoxide dismutase and Atrial Natriuretic Peptide (ANP) [271]. These mechanisms can aid in stopping the atrial electrical and structural remodeling that augments the likelihood of AF. In conclusion, the primary mechanisms by which metformin and TZDs can decrease the incidence of AF include reducing metabolic strain, insulin resistance, and inflammation, all of which are associated with the development of atrial fibrillation.

The mechanisms by which GLP-1RAs prevent AF differ and include stabilizing blood sugar, reducing epicardial fat, controlling body weight, managing calcium metabolism disorder, diminishing oxidative stress, and controlling blood pressure. GLP-1RAs contribute to weight loss by enhancing brown adipose tissue thermogenesis and the release of neuropeptide Y (NPY) and agouti-related peptide (AgRP). Additionally, GLP-1RAs lower levels of connective tissue growth factor (CTGF), which in turn reduces the production of reactive oxygen species (ROS) [273].

In contrast, the DPP-4 inhibitor alogliptin helps prevent AF through different mechanisms: it increases angiogenesis, promotes nitric oxide (NO) production, prevents mitochondrial membrane depolarization, enhances mitochondrial function, and stimulates mitochondrial biogenesis [274,275].

## 13. Renin–Angiotensin–Aldosterone System Modulating Medications

### 13.1. Angiotensin-Converting-Enzyme Inhibitors and Angiotensin Receptor Blockers

Recent studies indicate that angiotensin-converting enzyme inhibitors (ACEIs) and angiotensin receptor blockers (ARBs) might be especially valuable in treating AF, particularly among patients with common risk factors such as hypertension, left ventricular hypertrophy, chronic heart failure (cHF), and left ventricular dysfunction [276,277].

The renin–angiotensin–aldosterone system (RAAS) is associated with the pathophysiology of chronic inflammation as its mediators e.g., angiotensin II (AT2), which has been widely acknowledged to increase vascular permeability, recruit inflammatory cells, and promote their adhesion to the activated endothelium. Moreover, AT2 controls cell growth, regulates fibrosis, and augments ROS production, which leads to an increasing number of adhesion molecules, cytokines, and chemokines [278,279]. AT2 has also been widely acknowledged as an NFκB mediator [280], thus participating in NLPR3 inflammasome activation [281].

Furthermore, the renin–angiotensin–aldosterone system (RAAS) is crucial in the pathophysiology of heart remodeling. Therefore, angiotensin-converting enzyme inhibitors (ACEIs) and angiotensin receptor blockers (ARBs) can reverse and prevent it [282]. The RAAS components’ mechanisms that lead to heart remodeling include extracellular matrix deposition, myocyte hypertrophy, fibroblast proliferation, inflammatory immune cell activation, and hemodynamic alterations [283,284], which lead to eventual AF development [283].

A meta-analysis of ten studies, involving 42,892 patients, found that ACEIs/ARBs significantly reduced the incidence of AF recurrence when compared to calcium antagonists (*p* < 0.00001) or β-blockers (*p* = 0.005), respectively. However, this reduction was not observed in the incidence of new-onset AF [261]. In contrast, another meta-analysis of 23 RCTs, comprising 87,048 patients, showed ACEIs and ARBs’ effectiveness in primary prevention of AF in patients with heart failure (HF), hypertension, or left ventricular hypertrophy, but not in those who experienced a myocardial infarction [285]. Furthermore, a meta-analysis of 13,184 patients demonstrated similar outcomes in reducing AF recurrence between the ACEI and ARB groups (with an odds ratio of 0.42; 95% CI, 0.31−0.57) [286].

### 13.2. Mineralocorticoid Receptor Antagonists

Mineralocorticoid receptor antagonists (MRAs), including spironolactone and eplerenone, are widely utilized in the management of heart failure (HF) and hypertension. Beyond their established hypotensive effects, MRAs exert notable pleiotropism, such as antifibrotic and anti-inflammatory properties. Aldosterone, traditionally associated with ion and water transport regulation, promotes fibrosis and inflammation via mineralocorticoid receptor (MR) activation. This process triggers the transcription of profibrotic factors, such as PAI-1, TGF-β, PGF, CTGF, and collagen III [287]. Additionally, aldosterone fosters oxidative stress, amplifying the transcription of pro-inflammatory mediators, including NFκB, NLRP3, and Caspase-1. It also enhances lymphocyte activation and infiltration [288]. Moreover, aldosterone has been reported to exert an inflammatory effect independently of the mineralocorticoid receptor, which involves the phosphatidylinositol 3-kinase/Akt pathway [289]. Conversely, MRAs have been robustly proven to alter inflammation by inhibiting MRs [290,291,292,293].

Meta-analyses of RCTs have consistently reported a significant reduction in AF onset among patients with prescribed MRA treatment [294,295,296,297,298]. In a meta-analysis by Oraii et al. [298], MRAs have also been proven to reduce deaths related to cardiovascular death or HF hospitalization among patients with HF, regardless of the comorbid AF (HR = 0.95; 95% CI: 0.54–1.66). MRAs have also been proven to significantly reduce the risk of AF progression which has been emphasized in the meta-analysis by Rodrigues et al. [299] which mirrors the results on animal models. Nevertheless, as mentioned by Takemoto et al. [300], MRA cardioprotective properties have limitations as far as the non-inflammatory substrate of AF is concerned as, in their animal model, eplerenone did not alter the rate of dominant frequency and ion channel densities.

Unfortunately, as far as the relationship between POAF and MRAs is concerned, a meta-analysis evaluating the effect of MRAs on postoperative AF found no significant risk reduction (*p* = 0.09) [295]. Moreover, the IMPRESS-AF RCT [301] failed to prove the potential benefit of MRAs on exercise tolerance among patients with heart failure with preserved ejection fraction (HFpEF) and AF, as spironolactone did not improve peak oxygen consumption, mean 6-min walking distance, left ventricular diastolic function, or quality of life scores.

## 14. Targeted Anti-IL-6 Treatment

Interleukin-6 is one of the most potent endocrine-signaling pro-inflammatory molecules secreted by a wide variety of cells, including macrophages, osteoblasts, and smooth muscle cells in blood vessels; it is commonly associated with stimulating immunoglobulin production by promoting B-cell differentiation, T-cell proliferation [302], osteoclast maturation [303], and increasing oxidative stress. It is also involved in various autoimmune and chronic inflammatory diseases.

Over the years, IL-6 has been associated with the pathophysiology of numerous cardiological conditions. Direct measurements of circulating levels of IL-6, soluble IL-6 receptor, and soluble glycoprotein 130 (the IL-6 family of cytokines receptor), have confirmed a positive correlation between AF occurrence and the elevation of IL-6 trans-signaling level [28,304]. IL-6 rise is associated with the downregulation of cardiac connexins, which, in turn, leads to atrial electrical remodeling [28]. The exact role of Il-6 in the development of AF is still unknown. However, Il-6 has been reported to directly influence several cellular pathways involved in pathological activation of pro-inflammatory and profibrotic environments e.g., Xiao L et al. [305] have reported that Il-6-mediated activation of the JAK2/STAT3/Sox4 signaling pathway is involved in the activation of the NLRP3 inflammasome. Moreover, similarly to other JAK/STAT3 activators, Il-6 has been reported to stimulate the transcription of genes responsible for inflammation and fibrosis, including collagen, fibronectin, and α-SMA [306], which potentially plays a role in the fibrosis of the atrial myocardium.

However, until recently, researchers rarely focused on its correlation with the pathophysiology of AF. In 2020, Zhou et al. [307] conducted a meta-analysis of five studies exploring the connection between IL-6 and thromboembolic events in AF. High IL-6 was associated with a long-term stroke and thromboembolic risk (RR = 1.44, 95% CI 1.09–1.90, *p* = 0.01), long-term bleeding risk (RR = 1.36, 95% CI 1.06–1.74, *p* = 0.02), acute coronary syndrome (RR = 1.81, 95% CI 1.43–2.30, *p* < 0.001), as well as all-cause death (RR: 2.35, 95% CI: 2.09–2.65, *p* < 0.001).

IL-6 has also been shown to impact the risk of postoperative atrial fibrillation. In a prospective case–control study conducted by Tao et al. [308], higher levels of IL-6 were observed in patients who experienced the complication (92.29 vs. 63.40 pg/mL, *p* = 0.004). Moreover, the level of IL-6 after the surgery was associated with an increase in AF occurrence risk (OR = 1.02, 95% CI 1.01–1.04, *p* = 0.018). Usage of these results may help to ameliorate current prediction models, as well as increase the understanding of the pathogenesis underlying AF. Analogously, increased pre-procedure levels of IL-6 were highly indicative of a higher risk of AF recurrence after ablation [201,202,203]. Peripheral blood IL-6 level has also been demonstrated to increase after ablation, which may be associated with later incidences of thromboembolic complications [309]. Nevertheless, more research is needed to fully explore the optimal management of patients, post-ablation. When comparing balloon cryoablation and radiofrequency catheter ablation, IL-6 level results after the procedure were comparable between the groups, confirming previously established relations [310].

Similar correlations can be noticed in patients with cHF. An IL-6 rise can lead to an increase in the likelihood of AF (OR = 1.175, 95% CI 1.013–1.363, *p* = 0.034) [311]. The level of this interleukin is also associated with prolonged PR-segment duration in the case of severe COVID-19 [312], which may explain the origin of conduction impairment during the active phase of the disease. IL-6 was also shown to be connected to QT prolongation [313]; however, more data are needed, as only a few studies have focused on this subject.

Multiple anti-IL-6 medications are currently undergoing the process of clinical trials, nonetheless, the ones approved first (e.g., tocilizumab) have already patched their way into cardiology [209,210,211]. This was mostly archived by the great amount of data gained from patients already treated with these drugs for other conditions, such as COVID-19.

More recently, ziltivekimab has gained a fair amount of attention, as it was established as possibly effective in the reduction of cardiovascular events [314,315,316]. Thus far, studies on ziltivekimab in AF have not been published. One of the studies, crucial to understanding the potential associated with ziltivekimab, has proven it to induce the reduction of neutrophil-to-leukocyte ratio [215] which has been associated with multiple inflammatory heart diseases [317] and is a plausible predictor of mortality in the general population (HR = 1.14, 95% CI 1.10–1.17) [318].

Optimism about blocking IL-6 pathways was further aggravated by their potential to reverse myocardial remodeling; nevertheless, initial studies were only conducted on post-ischemic changes [223,224]. IL-6 was furthermore proposed as a biomarker for left ventricle remodeling [220]. Indeed, the interleukin possesses the capacity to promote the depletion of NLRC5, a crucial regulator of adaptive immune response [319,320], shown to protect from adverse remodeling.

## 15. Discussion

Atrial fibrillation is inherently an incurable disease, management of which still poses a challenge for medical practitioners, due to its highly variable course among patients and the fact that available therapeutic strategies seem not to address its underlying pathophysiological drivers fully, nor reverse their impact on the cardiac electrophysiology. According to current evidence, the main pro-arrhythmic consequences of inflammation include enhanced fibrosis, myocardial remodeling, and electrophysiological alterations due to the immune activity of various cells. Several studies have also established a correlation between some inflammatory diseases and pro-inflammatory biomarkers’ activity with AF progression.

**Figure 4:**
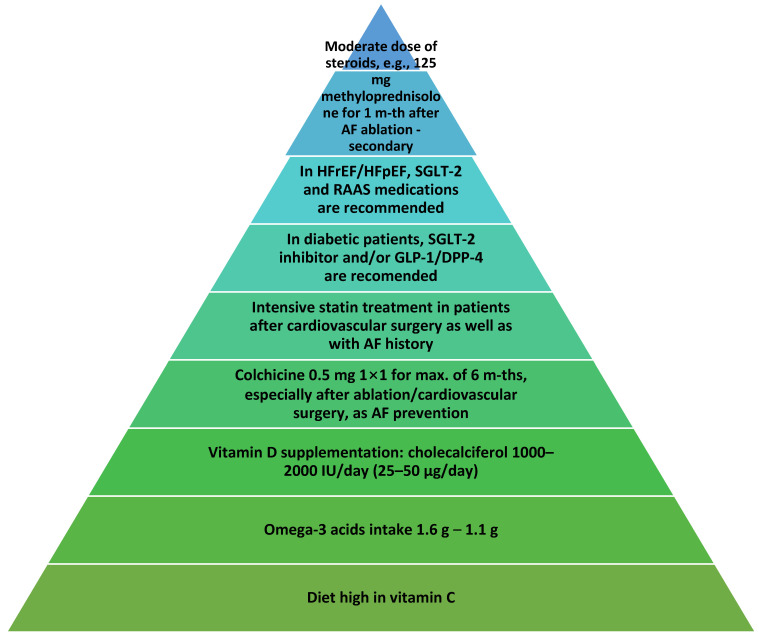
A flowchart depicting a sequential algorithm that clinicians can use as simplified guidelines for managing patients with AF.

Unfortunately, currently known methods of immunomodulation do not significantly reduce the risk of AF occurrence, at best decreasing it by a marginal percentage. Moreover, randomized controlled trials face the challenge of establishing a correlation between drugs with documented anti-inflammatory properties and their potential benefits for patients with AF, due to the difficulty in measuring systemic inflammation and assembling a sufficiently homogenous group of patients with similar disease profiles and levels of systemic inflammation. Nevertheless, several real-life trials have congenitally provided evidence that the use of statins and other potent anti-inflammatory drugs result in beneficial effects in patients with AF, such as a lowered risk of stroke [215], a decreased rate of AF recurrence, and alleviation of cardiac dysfunction. However, it does not seem to slow down the disease progression itself [321]. The most promising results come from research concerning ACEIs, ARBs, and MRAs, showing that they significantly reduce the risk of both new-onset and recurrent AF. This might be due to their pleiotropic effects, such as preventing and inhibiting the inflammatory reactions induced by RAA pathway mediators. Table 1 presents selected, relevant studies [Table 1]. We offer a straightforward sequential algorithm derived from clinical trials that clinicians can use as simplified guidelines for managing patients with AF [Figure 4].

While we still need more data to evaluate the safety and efficacy of anti-inflammatory polypharmacotherapy thoroughly, some of the mentioned drugs have already been extensively combined to treat various diseases in clinical practice. Several studies have examined potential interactions and adverse effects of these combinations, providing promising results [322,323]. However, additional research focusing on their impact on inflammation and AF is crucial for the evaluation of their additive effect and benefits.

Further research investigating the clinical utility of experimental targeted treatment on the NLRP3 inflammasome e.g., MCC950, dapansutrile, RRx-001, DFV890, Bay 11-7082, Βeta-hydroxybutyrate, usnoflast, ibrutinib, and CY-09, or drugs targeting crucial checkpoints of the NLRP3 inflammasome-pathway e.g., Caspase-1 inhibitors (VX-765 and VX-740) or GSDMD inhibitors (disulfiram and necrosulfonamide), might potentially bring hope for the better management of AF. However, currently, most of these drugs are still in their preclinical phase [324].

**Table 1:** The key studies on the effect of anti-inflammatory drugs on atrial fibrillation.

	Mechanism	Primary Use	Key Clinical Trials	Effect
Nonsteroidal anti-inflammatory drugs	Inhibition of cyclooxygenase (COX) enzymes; inhibition of prostaglandin synthesis causes fluid retention	Analgesics and anti-inflammatory treatments	Pizzuto K et al. [113] (cross-sectional, prospective)	Positive
Chloroquine, hydroxychloroquine	Prolongation of an action potential duration that leads to a cease of arrhythmia in the mechanism of re-entrant excitation	Antimalarial drugs	Filgueiras-Rama D et al. [131] (prospective, comparative trial)	Positive
Antioxidants, e.g., quercetin;	Inhibition of lipid peroxidation; modulation of the MAPK signaling pathway; interference with the NFκB/TNFα pathway	A nutritional supplement; a regular component of a normal diet	Li D et al. [138] (prospective trial)	Positive
Ascorbic acid (vitamin C)			Trankle CR et al. [161] (prospective, randomized, double-blinded, placebo-controlled)	Positive
			Dehghani MR et al. [162] (prospective, non-randomized study)	Positive
Anti-IL-6 monoclonal antibody-tocilizumab	Reduction in the QTc interval duration	Inflammatory and autoimmune disorders; in adults with COVID-19 receiving systemic corticosteroids and supplemental oxygen or mechanical ventilation	Lazzerini PE et al. [28] (prospective, non-control trial)	Positive
Il-1β (interleukin 1β)—Canakinumab	Paracrine signaling of the inflammatory cascade	Cryopyrin-associated periodic syndrome, Muckle–Wells syndrome, familial cold auto-inflammatory syndrome	Krisai P et al. [140] (prospective, randomized trial)	Neutral
Glucocorticosteroids	Inhibiting the production of reactive oxygen species (ROS); actively suppressing the release of interleukin-1 beta (IL-1β) and interleukin-18 (IL-18) through direct inhibition of the NLRP3 inflammasome; inhibition of the release of histamine from mast cells	Autoimmune disorders and hematological diseases	Iskandar S et al. [156] (prospective, randomized)	Neutral
Colchicine	Inhibition of ROS production; active suppression of the release of IL-1-β and IL-18 through direct inhibition of the NLRP3 inflammasome; inhibition of the release of histamine from mast cells	Gout flares; Familial Mediterranean Fever (FMF), Behçet’s syndrome, pericarditis, and postcardiotomy syndrome	Deftereos s et al. [325] (prospective, randomized control study)	Positive
Statins	Reversible competitive inhibition of HMG-CoA reductase; impairment of the mevalonate pathway; sphingosine-1-phosphate pathway activation; increase in Ca^2+^ concentration and further promotion of Ca^2+^-signaling pathways involving pathological overactivation of cardiac type 2 ryanodine receptors; activation of the Akt/Nrf2/HO-1 pathway, which delivers heme oxygenase-1 (HO-1)—structural remodeling suppression		Albert MA et al. [186] (prospective, randomized control study)	Positive
Omega-3 polyunsaturated fatty acids	Reduction in thromboxane A2 activity; antioxidative properties; decreasing substantially pro-inflammatory cytokines such as Il-6 and TNF-alfa; downregulation of nuclear factor kappa B (NF-κB), an essential mediator of the NLRP3 inflammasome; regulatory function of Ca^2+^ ion distribution within the cardiomyocytes; the upregulation of the Cx43	Reducing triglyceride levels in blood serum, decreasing blood pressure, and potent anti-inflammatory properties	Bhatt DL et al. [234] (prospective, randomized, double-blind, placebo-controlled) Nicholls SJ et al. [235] (prospective, randomized, double-blind)	Positive
Vitamin D	Attenuation of IFN-γ, TNF-α, IL-1β, IL-6, IL-8, IL-12, and IL-17 secretion, and increased anti-inflammatory cytokines IL-4 and IL-10; regulation of the immune system directly via VDRs (vitamin D receptors); decrease in NLRP3 gene expression and activation, inhibiting further excretion of profibrotic cytokines	A nutritional supplement	Virtanen JK et al. [248] (randomized, double-blind, placebo-controlled)	Positive
Antidiabetic Drugs - sodium–glucose cotransporter-2 (SGLT2) inhibitors	Affect AMP-activated protein kinase (AMPK); decrease pro-inflammatory cytokines IL-6, IL-1beta, and TNF-alpha production and reduce the M1/M2 macrophages ratio	Diabetes Melitus; Heart Failure; Chronic Kidney Disease	Zelniker TA et al. [258] (prospective, non-randomized)	Positive
Glucagon-like peptide-1 receptor agonists (GLP-1RAs)—thiazolidinediones	Peroxisome proliferator-activated receptor gamma (PPARγ) activators; regulation of signal transduction pathways engaging tumor necrosis factor- α (TNF-α), transforming growth factor-β (TGF-β), superoxide dismutase, and Atrial Natriuretic Peptide (ANP); lowering connective tissue growth factor (CTGF) levels, reducing the production of reactive oxygen species (ROS)		Zhang X et al. [274] (prospective, randomized)	Positive
DPP-4 inhibitor	Promotes nitric oxide (NO) production, prevents mitochondrial membrane depolarization, enhances mitochondrial function, and stimulates mitochondrial biogenesis		Yamamoto T et al. [275] (prospective, randomized, animal model study)	Positive

## Data Availability

No new data were created or analyzed in this study. Data sharing is not applicable to this article.
